# Predicting Patients’ Intention to Use a Personal Health Record Using an Adapted Unified Theory of Acceptance and Use of Technology Model: Secondary Data Analysis

**DOI:** 10.2196/30214

**Published:** 2021-08-17

**Authors:** Consuela Cheriece Yousef, Teresa M Salgado, Ali Farooq, Keisha Burnett, Laura E McClelland, Abin Thomas, Ahmed O Alenazi, Laila Carolina Abu Esba, Aeshah AlAzmi, Abrar Fahad Alhameed, Ahmed Hattan, Sumaya Elgadi, Saleh Almekhloof, Mohammed A AlShammary, Nazzal Abdullah Alanezi, Hani Solaiman Alhamdan, Sahal Khoshhal, Jonathan P DeShazo

**Affiliations:** 1 Pharmaceutical Care Department Ministry of National Guard-Health Affairs Dammam Saudi Arabia; 2 King Abdullah International Medical Research Center Riyadh Saudi Arabia; 3 King Saud bin Abdul-Aziz University for Health Sciences Riyadh Saudi Arabia; 4 Department of Pharmacotherapy & Outcome Science School of Pharmacy Virginia Commonwealth University Richmond, VA United States; 5 Department of Computing University of Turku Turku Finland; 6 Department of Clinical Laboratory Sciences Cytopathology Practice Program University of Tennessee Health Science Center Memphis, TN United States; 7 Department of Health Administration Virginia Commonwealth University Richmond, VA United States; 8 Department of Biostatistics and Bioinformatics King Abdullah International Medical Research Center Riyadh Saudi Arabia; 9 Pharmaceutical Care Department Ministry of National Guard-Health Affairs Riyadh Saudi Arabia; 10 Pharmaceutical Care Department Ministry of National Guard-Health Affairs Jeddah Saudi Arabia; 11 Pharmaceutical Care Department Ministry of National Guard-Health Affairs Madinah Saudi Arabia; 12 Department of Pharmacy Practice College of Pharmacy Princess Noura Bint Abdulrahman University Riyadh Saudi Arabia; 13 Pharmaceutical Care Department Ministry of National Guard-Health Affairs Al Ahsa Saudi Arabia; 14 Primary Health Care Prince Bader Housing Clinic Riyadh Saudi Arabia; 15 Qassim Primary Health Care Center Ministry of National Guard-Health Affairs Qassim Saudi Arabia

**Keywords:** personal health record, patient portal, eHealth, Middle East, Saudi Arabia, Unified Theory of Acceptance and Use of Technology, prediction, intention, electronic health record, acceptance, model, framework, secondary analysis

## Abstract

**Background:**

With the rise in the use of information and communication technologies in health care, patients have been encouraged to use eHealth tools such as personal health records (PHRs) for better health and well-being services. PHRs support patient-centered care and patient engagement. To support the achievement of the Kingdom of Saudi Arabia’s Vision 2030 ambitions, the National Transformation program provides a framework to use PHRs in meeting the 3-fold aim for health care—increased access, reduced cost, and improved quality of care—and to provide patient- and person-centered care. However, there has been limited research on PHR uptake within the country.

**Objective:**

Using the Unified Theory of Acceptance and Use of Technology (UTAUT) as the theoretical framework, this study aims at identifying predictors of patient intention to utilize the Ministry of National Guard-Health Affairs PHR (MNGHA Care) app.

**Methods:**

Using secondary data from a cross-sectional survey, data measuring the intention to use the MNGHA Care app, along with its predictors, were collected from among adults (n=324) visiting Ministry of National Guard-Health Affairs facilities in Riyadh, Jeddah, Dammam, Madinah, Al Ahsa, and Qassim. The relationship of predictors (main theory constructs) and moderators (age, gender, and experience with health apps) with the dependent variable (intention to use MNGHA Care) was tested using hierarchical multiple regression.

**Results:**

Of the eligible population, a total of 261 adult patients were included in the analysis. They had a mean age of 35.07 (SD 9.61) years, 50.6 % were male (n=132), 45.2% had university-level education (n=118), and 53.3% had at least 1 chronic medical condition (n=139). The model explained 48.9% of the variance in behavioral intention to use the PHR (*P*=.38). Performance expectancy, effort expectancy, and positive attitude were significantly associated with behavioral intention to use the PHR (*P*<.05). Prior experience with health apps moderated the relationship between social influence and behavioral intention to use the PHR (*P*=.04).

**Conclusions:**

This study contributes to the existing literature on PHR adoption broadly as well as in the context of the Kingdom of Saudi Arabia. Understanding which factors are associated with patient adoption of PHRs can guide future development and support the country’s aim of transforming the health care system. Similar to previous studies on PHR adoption, performance expectancy, effort expectancy, and positive attitude are important factors, and practical consideration should be given to support these areas.

## Introduction

### Background

The transformation of health care delivery has been a global phenomenon since the turn of the 21st century [1,2]. Health care delivery has evolved from a paternalistic “doctor knows best” model to one where individuals are encouraged to play an active role in their health [3]. As the prevalence of chronic diseases increases along with the rise in information and communication technologies, patients have been encouraged to accept more responsibility for their health and well-being by using eHealth tools [4,5].

Personal health records (PHRs) are eHealth tools that aim to increase patient engagement and empowerment by allowing individuals to keep track of their personal health information. PHRs have been defined as “an Internet-based set of tools that allows people to access and coordinate their lifelong health information and make appropriate parts of it available to those who need it” [6]. Nevertheless, PHR has no uniform definition, with numerous terms being used interchangeably in the literature, namely “patient web portal,” “patient portal,” “computerized patient portal,” “patient-accessible electronic health record,” “tethered PHR,” and “electronic PHR.” PHRs hold great potential in chronic disease management [7].

Health care organizations adopt PHRs to increase patient engagement to meet the 3-fold aim for health care: increased access, reduced cost, and improved quality of care [7-9]. Some of the proposed benefits from the use of PHRs are empowerment, continuity of care, education, patient-provider partnership, individual control, and engagement. Managing chronic diseases requires regular use of self-management skills such as identifying problems, finding solutions, using information sources, collaborating with health care providers, altering behavior, and assessing results [10].

### Research Problem and Aim

In 2018, the Ministry of National Guard Health Affairs (MNG-HA) implemented its PHR, known as MNGHA Care. MNHGA Care features include checking laboratory results, scheduling appointments, requesting medical reports, requesting prescription refills, viewing radiology reports, and providing vaccination reminders. It allows patients to upload personal health information such as blood pressure, blood sugar measurements, weight, and exercise information. A self-assessment feature allows patients to enter information on pain control, performance status, and quality of life. Educational resources are also provided on the PHR. Two years prior to implementing the PHR, Al Sahan and Saddik [11] evaluated the knowledge and perceptions toward using a PHR among 454 patients and 9 technical staff from an MNG-HA hospital in Riyadh before implementation. Participants reported a high level of interest (very interested: 60.6%, interested: 25.2%) in a web-based PHR. Since the implementation, further research is needed on patient adoption.

The aim of this study was to identify a set of constructs that predict the intention to use the MNGHA Care PHR among patients, using the Unified Theory of Acceptance and Use of Technology (UTAUT) as a theoretical framework. Before a technology is adopted, a user must first intend to use the technology [12]. The benefits of increased accessibility, reduced costs, and better quality of health care with the PHR can only be achieved by understanding what motivates individuals to use this technology.

### Theoretical Framework

While there are many models available to explain user acceptance, Venkatesh et al [12] developed the UTAUT to provide a comprehensive framework to explain acceptance and usage of information technology in organizations. It is a synthesis of 8 theoretical models, including Theory of Reasoned Action, Technology Acceptance Model, Motivational Model, Theory of Planned Behavior, Combined Technology Acceptance Model–Theory of Planned Behavior, Model of Personal Computer Utilization, Diffusion of Innovation Theory, and Social Cognitive Theory [12]. Venkatesh et al [12] evaluated the independent variables that influence behavioral intention and actual use of technology. The three independent constructs—performance expectancy, effort expectancy, and social influence—directly influence the behavioral intention to use technology. Facilitating conditions and behavioral intention act directly on actual use of technology. Gender, age, voluntariness, and experience are moderators in the framework. This study will adapt UTAUT to investigate the factors that influence patients’ intention to use MNGHA Care.

**Figure 1:**
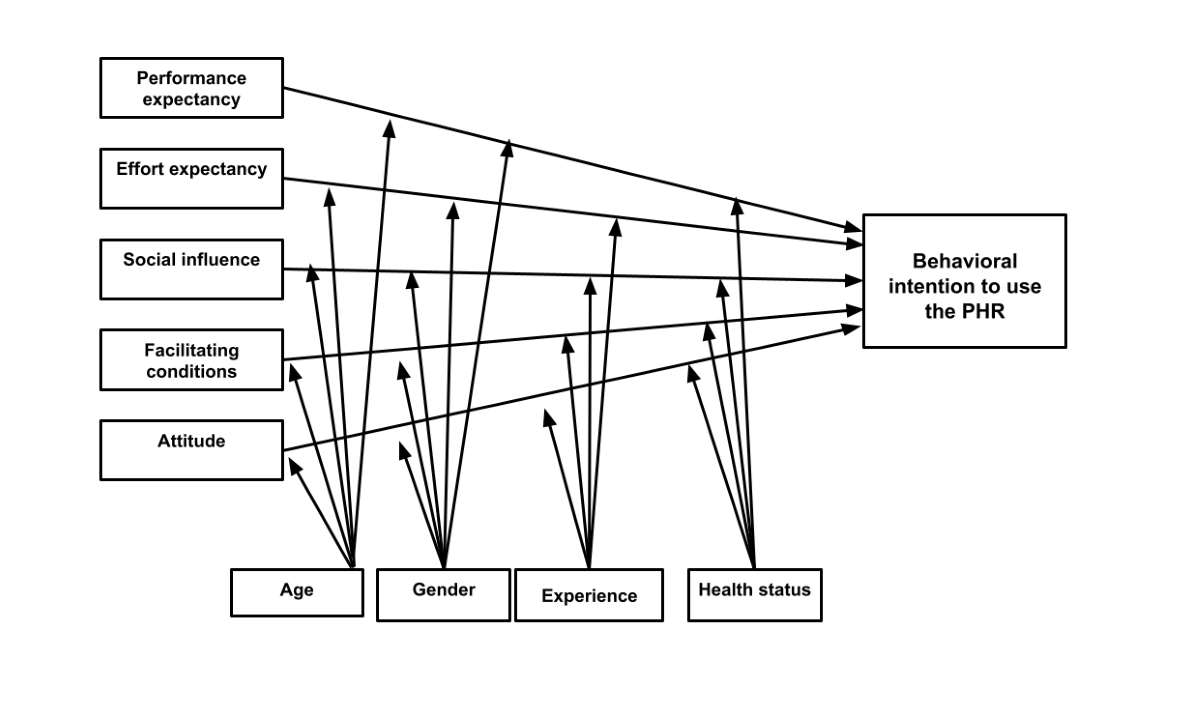
Adapted Unified Theory of Acceptance and Use of Technology model to predict patient intention to use the MNGHA Care PHR.

**Figure 2:**
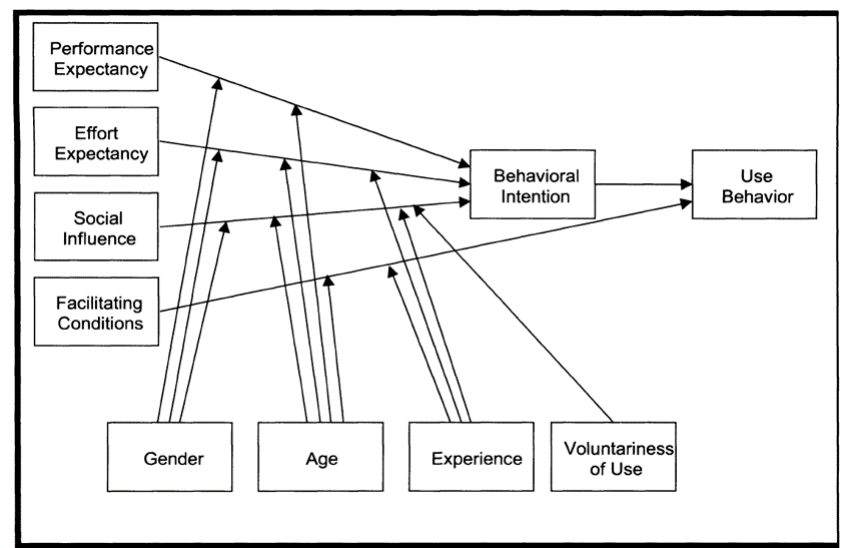
Original Unified Theory of Acceptance and Use of Technology [12].

The adapted UTAUT model for this study is presented in Figure 1. Figure 2 shows the original UTAUT. There are 3 adaptations to the original model. First, the construct of attitude is added. In the critical review of the UTAUT model, Dwivedi et al [13] recommended revising the model to include the construct of attitude. Individual characteristics are not included in UTAUT [13]. However, studies have found individual traits to be important predictors of technology acceptance [14,15]. Secondly, the moderators of gender, age, experience, and voluntariness of use are used in the original UTAUT model. In the adapted model, the voluntariness of use is dropped as a moderator since PHR use is voluntary. Finally, health status is added to moderate the relationships among the predictors and the behavioral intention to use the PHR.

The proposed differences between this research model and the original UTAUT model are shown in Table 1. Age and gender will moderate all relationships. Women and younger individuals are expected to have a stronger behavioral intention to use the PHR. Experience is operationalized as the prior use of health apps. Venkatesh et al [12] characterized experience as experience with the system being implemented. Experience using a health app would imply that the individual has the necessary computer and internet skills to use a PHR. Limited computer and internet experience has been identified as a barrier to PHR adoption [16]. Individuals with experience using health apps are expected to have a stronger behavioral intention to use the PHR. Finally, health status was selected as a moderator because it has been shown to be an important driver of PHR acceptance [8,17]. If resources and support are available, individuals with poorer health are more likely to use eHealth technologies [18]. Health status in this study will be based on self-reported health status. Patients with poorer health status are expected to have a stronger behavioral intention to use the PHR.

**Table 1:** Proposed differences between the original and adapted Unified Theory of Acceptance and Use of Technology model for patients.

Relationships	Original model moderators				Adapted model moderators			
	Gender	Age	Experience	Voluntariness	Age	Gender	Experience	Health status
Performance expectancy–behavioral intention	✓	✓			✓	✓		✓
Effort expectancy–behavioral intention	✓	✓	✓		✓	✓	✓	
Social influence–behavioral intention	✓	✓	✓	✓	✓	✓	✓	✓
Behavioral intention–actual usage								
Facilitating conditions–actual usage		✓	✓					
Facilitating conditions–behavioral intention					✓	✓	✓	✓
Attitude–behavioral intention					✓	✓	✓	✓

## Methods

### Study Design

Data for the study were obtained from a cross-sectional survey study [19] in which data were collected to examine health information–seeking behavior and PHR (MNG-HA Care) use among patients. Secondary data were used in the current study. Institutional Review Board approval (RD19/002/D) was obtained from King Abdullah International Medical Research Center and Virginia Commonwealth University (HM20020713).

### Setting and Participants

MNG-HA is a large health care system that provides medical care to the National Guard’s soldiers and their dependents in all regions across the Kingdom of Saudi Arabia. The target study population consisted of adults who visited outpatient facilities (primary or specialty care) in five major cities—Dammam, Riyadh, Jeddah, Madinah, and Qassim. In the original study, a total of 546 adults completed the survey.

For this secondary analysis, participants who answered all questions related to the use of the MNGHA Care PHR constituted the study sample (n=324). A minimum sample size of 270 was calculated for the analysis on the basis of the 10 times rule, which posits that the minimum sample size should be 10 times the number of predictors (27 in this case, including 5 independent variables, 4 moderators, and 18 interaction terms) [20].

### Data Collection

As mentioned above, secondary data were used in this study. The original data were collected between December 2019 and February 2020. The survey we used was adapted from Hoogenbosch et al [21]’s study of a PHR using UTAUT, with minor modifications to existing items and additional items created to fit the objectives of the study. Responses to each question were provided on a 5-point Likert scale from 1 (strongly disagree) to 5 (strongly agree). However, questions were limited to avoid respondent burden resulting in 1 or 2 items used for each construct.

Behavioral intention, the dependent variable, measures the strength of an individual’s intention to perform a specific behavior; that is, to use the MNGHA Care PHR [22]. A 2-item scale was used to measure behavioral intention: “I will probably use MNGHA Care in the future” and “I intend to use MNGHA Care regularly.” The reliability coefficient was Cronbach α=.76.

Performance expectancy reflects the degree to which an individual believes that using a technology will help attain significant rewards. Unlike Hoogenbosch et al [21], who used 3 items to measure this construct, we used the single item, “By using MNGHA Care, I feel more involved in my care.”

Effort expectancy is the degree of ease associated with the use of technology—in this case, the PHR [12]. The single item, “Information in MNGHA Care is understandable,” was used to measure effort expectancy, unlike Hoogenbosch et al [21], who used a 5-item scale.

Social influence refers to an individual’s perception of how important people in their social circle are, using technology [12]. Consistent with Hoogenbosch et al [21], the following item was used to measure this construct: “My healthcare professional encouraged me to use MNGHA Care.”

The construct of facilitating condition refers to organizational and technical infrastructure support technology use [12]. The single item, “Technical help is available when I do not know how to use MNGHA Care,” was used to measure this construct instead of the 3 items used by Hoogenbosch et al [21].

Attitude relates to positive or negative feelings associated with using a technology [22] and was assessed with the self-constructed item “MNGHA Care is a valuable service.”

Performance expectancy, effort expectancy, social influence, facilitating conditions, and attitude were independent variables.

Self-reported age, educational level, gender, health care facility, marital status, employment status, and monthly household income were recorded. Health care characteristics included the following: presence of a medical condition, number and type of medical conditions, self-reported health status, hospitalization in the past 6 months, and emergency department visits in the past 6 months. Health status was a categorical variable self-reported as excellent, very good, good, fair, or poor. Experience was a dichotomous variable defined as experience with health apps and assessed through the question: “Do you use health applications (apps) on your mobile phone?”

The moderators for the model were age, gender, experience, and health status.

### Statistical Analysis

Descriptive statistics and hierarchical multiple regression were conducted using SPSS (version 25, IBM Corp) [23]. While structural equation modeling is a more robust statistical method for testing a theoretical model and allows for single-item measures [24], it was not used owing to concerns that the model would not yield good results since all constructs were a single item. Data were assessed for normality, linearity, homoscedasticity, and absence of multicollinearity. Normality was assessed using skewness and kurtosis and found to be within the required threshold of –1.96 to +1.96 [25]. A Kolmogorov–Smirnov test was also used to test for normality with nonstatistical significance (*P*>.05), indicating that the data were normally distributed. Independence of observations was tested using the Durbin–Watson test, which yielded a coefficient of 1.905. As a rule of thumb, values between 1.5 and 2.5 are considered normal [26]. Linearity was confirmed by the appearance of a linear representation of standardized residuals on a scatterplot. Multicollinearity was checked by examining correlations and variance inflation factor (VIF) between variables. A VIF above 10 is an indicator of multicollinearity [27]. "No VIF greater than 10 was identified, indicating a lack of multicollinearity.

Three-stage hierarchical multiple regression analysis was conducted with behavioral intention as the dependent variable. The independent variables were entered into the regression model in 3 sequential blocks with all assumptions of regression met and outliers removed. The first block included the 5 independent variables of performance expectancy, effort expectancy, social influence, facilitating conditions, and attitude. The second block contained the moderator variables of age, gender, experience, health status, and independent variables. Experience was a categorical variable with 0 representing people with no experience using health apps and 1 representing people with experience using health apps. To test the moderating effects of gender, age, experience, and health status on the relationship of independent variables (performance expectancy, effort expectancy, social influence, facilitating conditions, and attitude) and behavioral intention to use the PHR, interaction terms were added to the regression model in block 3. For each block, the standardized regression coefficient (β) and the *R*^2^ were calculated.

## Results

### Demographic and Health Care Characteristics

Of the 324 participants who completed the survey about MNGHA Care use, 261 comprised the final sample after outlier removal. The mean age of the participants was 35.07 (SD 9.61) years. Most users were male (n=132, 50.6%), from the Central region (n=110, 42.1%), married (n=208, 79.7%), and had a higher educational level (university graduate: n=118, 45.2%) and a monthly income of at least US $2666 (n=95, 36.4%). For health status, the majority of participants (n=178, 68.2%) had a medical condition with the following being the most common chronic conditions: asthma or chronic obstructive pulmonary disease (n=46, 17.6%), diabetes (n=38, 14.6%), and hypertension (n=32, 12.3%). Table 2 summarizes the demographic and health care characteristics of the respondents.

**Table 2:** Demographic and health care characteristics of the study participants (N=261).

Characteristic			Value
**Demographic information**			
	Age (years), mean (SD)		35.07 (9.61)
	**Region of the country, n (%)**		
		Eastern	81(31.0)
		Central	110 (42.1)
		Western	70 (26.8)
	**Gender, n (%)**		
		Male	132 (50.6)
		Female	129 (49.4)
	**Marital status, n (%)**		
		Married	208 (79.7)
		Single	53 (20.3)
	**Education level, n (%)**		
		Elementary school or less	14 (5.4)
		Middle school	17 (6.5)
		High school	91 (34.9)
		University	118 (45.2)
		Postgraduate	20 (7.7)
	**Employment status, n (%)**		
		Employed	142 (54.4)
		Retired	16 (6.1)
		Student	17 (6.5)
		Unemployed	84 (32.2)
	**Monthly household income, n (%)**		
		<5000 SAR (US $1333)	69 (26.4)
		5000-9999 SAR (US $1333-2666)	84 (32.2)
		>10,000 SAR (US $2666)	95 (36.4)
**Health status characteristics**			
	Have a medical condition, n (%)		178 (68.2)
	**Number of medical conditions, n (%)**		
		None	83 (31.8)
		1	139 (53.3)
		≥2	39 (14.9)
	**Type of medical condition, n (%)**		
		Diabetes	38 (14.6)
		Hypertension	32 (12.3)
		Asthma or chronic obstructive pulmonary disease	46 (17.6)
		Heart failure	9 (3.4)
		Cancer	11 (4.2)
		Sickle cell disease	7 (2.7)
		Psychiatric condition	4 (1.5)
		Other	78 (29.9)
	**Self-reported health status, n (%)**		
		Excellent	121 (46.4)
		Very good	95 (36.4)
		Good	33 (12.6)
		Fair	8 (3.1)
		Poor	4 (1.5)
	Hospitalized within the last 6 months, n (%)		54 (20.7)
	Visited the emergency department within the last 6 months, n (%)		124 (47.5)

### Hypothesized Relationships

The results of hierarchical multiple regression analysis are presented in Table 3. The first stage of the model revealed that performance expectancy, effort expectancy, social influence, facilitating conditions, and attitude contributed significantly to the regression model (*F*_5,255_=38.874; *P*<.001) and accounted for 43.3% of the explained variance in patients’ intention to use MNGHA Care. Effort expectancy and attitude were almost equally important predictors with standardized regression coefficients of 0.249 and 0.198, respectively.

In the second stage of the model, the variables age, gender, experience with health applications, and health status were entered along with the independent variables. These variables did not significantly contribute to the regression model with an additional explained variance of 0.8% in the *R*^2^ value (*F*_4,251_=0.950; *P*=.44). 

**Figure 3:**
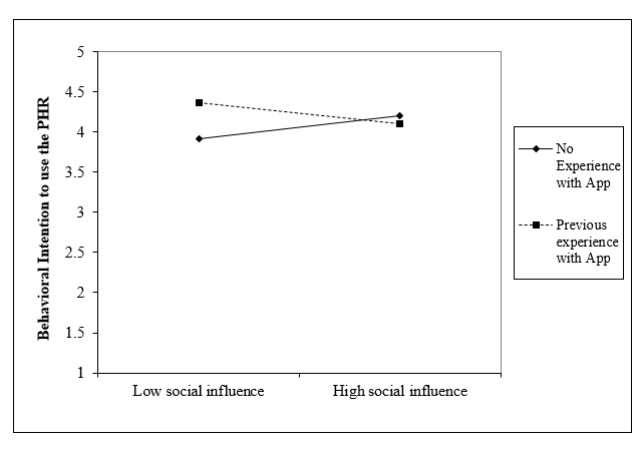
Interaction between social influence and experience on behavioral intention (*P*=.04). PHR: personal health record.

In the third stage, the full model included the independent variables, moderating variables (age, gender, experience with health applications, and health status), and interaction terms. Adding the interaction terms to the model accounted for an additional 5.6% of explained variance and was not significant (*F*_20,231_=1.075; *P*=.38). Figure 3 reflects the moderating effect of app experience on social influence in behavioral intention to use the PHR (β=–0.236; t_231_=–2.036; *P*=0.04).

**Table 3:** Summary of the results of hierarchical regression analysis for variables predicting behavioral intention to use the personal health record among study participants (N=261).

Variables		β (SE)	β	*t* test (*df*)	*R* ^2^
**Block 1**					0.433^b^
	Performance expectancy	0.261^a^ (0.054)	0.286^a^	4.847^a^ (*255*)	
	Effort expectancy	247^a^ (0.057)	0.249^a^	4.338^a^ (*255*)	
	Social influence	011 (0.040)	0.017	0.267 (*255*)	
	Facilitating conditions	062 (0.038)	0.100	1.618 (*255*)	
	Attitude	0.174^a^ (0.053)	0.198^a^	3.282^a^ (*255*)	
**Block 2**					0.441
	Gender	–0.008 (.055)	–0.007	–0.150 (*251*)	
	Age	0.001 (.003)	0.023	0.446 (*251*)	
	Experience	0.108 (.056)	0.095	1.934 (*251*)	
	Health status	–0.003 (.030)	–0.004	–0.084 (*251*)	
**Block 3**					0.489
	Performance expectancy * gender	0.184 (0.126)	0.143	1.456 (*231*)	
	Performance expectancy * age	0.005 (0.009)	0.052	0.631 (*231*)	
	Performance expectancy * experience	0.128 (0.129)	0.116	0.991 (*231*)	
	Performance expectancy * health status	0.034 (0.083)	0.034	0.403 (*231*)	
	Effort expectancy * gender	–0.131 (0.129)	–0.099	–1.013 (*231*)	
	Effort expectancy * age	–0.006 (0.007)	–0.053	–0.875 (*231*)	
	Effort expectancy * experience	0.027 (0.141)	0.022	0.191 (*231*)	
	Effort expectancy * health status	0.001 (0.061)	0.001	0.018 (*231*)	
	Social influence * gender	0.064 (0.088)	0.071	0.734 (*231*)	
	Social influence * age	–0.005 (0.005)	–0.066	–0.912 (*231*)	
	Social influence * experience	–0.182^a^ (0.090)	–0.236^a^	–2.036^a^ (*231*)	
	Social influence * health status	–0.079 (0.054)	–0.107	–1.459 (*231*)	
	Facilitating conditions * gender	0.016 (0.091)	0.020	0.178 (*231*)	
	Facilitating conditions * age	–0.002 (0.004)	–0.038	–0.506 (*231*)	
	Facilitating conditions * experience	0.074 (0.093)	0.096	0.794 (*231*)	
	Facilitating conditions * health status	0.002 (0.055)	0.003	0.043 (*231*)	
	Attitude * gender	–0.115 (0.124)	–0.098	–0.930 (*231*)	
	Attitude * age	–0.008 (0.008)	–0.080	–1.015 (*231*)	
	Attitude * experience	–0.219 (0.144)	–0.206	–1.518 (*231*)	
	Attitude * health status	0.034 (0.082)	0.035	0.421 (*231*)	

^a^*P*<.05.

^b^*P*<.001.

## Discussion

### Principal Findings

This study attempted to identify predictors in the adoption of the MNGHA Care PHR among patients from a single, large, integrated health care organization in the Kingdom of Saudi Arabia, using an adapted UTAUT model. The structural model used in this study explained 48.9% of the variance in behavioral intention to use MNGHA Care. Performance expectancy, effort expectancy, and positive attitude were positive predictors of behavioral intention, confirming the construct of attitude has a significant impact on PHR adoption. The individual characteristics of age, gender, experience with health applications, and health status did not significantly influence behavioral intention. As depicted in Figure 3, higher social influence led to higher behavioral intention to use MNGHA Care in patients without previous experience using health apps. On the contrary, among patients who had experience using health applications, social influence negatively affected behavioral intention to use the app. There was a greater impact of experience with low social influence than with the high social influence.

Other studies have also shown performance expectancy and effort expectancy to be significantly and positively associated with PHR adoption [8,18,21,28-31]. This study supports the evidence that patients are more likely to use PHRs when they perceive them as useful and easy to use.

In this study, social influence and facilitating conditions were not associated with behavioral intention. This aligns with the findings of Tavares and Oliveira [18]. Although social influences such as interactions with health care providers have been identified as important in patients’ adoption of PHRs, our findings did not find a significant impact [7,8,32]. Yousef et al [19], however, reported that health care providers (47.9%) or hospital staff (10.8%) were mainly responsible for recommending the use of MNGHA Care. Facilitating conditions likely did not have a significant impact as users found the organizational resources and technical help adequate.

Finally, a positive attitude toward the PHR was found to have a significant impact on behavioral intention. Attitude is a strong predictor of behavioral intention to use various types of technology and is the direct precedent of intention [22]. This is aligned with the findings of other studies on PHRs [28,33]. Since attitudes may be influenced by various factors (eg, peers, health care providers, and other health care staff), promoting the PHR can encourage positive attitudes, which can ultimately lead to PHR adoption.

### Implications for Theory

This study contributes to the existing literature on PHRs and provides several implications for theory. First, it provides an understanding of the predictors of PHR adoption in general and, more specifically, within the context of the Middle East and the Kingdom of Saudi Arabia. PHRs have not been widely adopted, and there is limited data on predictors of PHR adoption in this region [34,35].

Second, it extends UTAUT with the construct of attitude and the moderators “experience with health applications” and “health status” in a health care setting. The results of this study provided further support for the constructs of performance expectancy, effort expectancy, and attitude to have significant and positive effects on PHR adoption, which is consistent with the literature. Alsahafi et al [34] was the first study to empirically examine predictors of PHR acceptance in the Kingdom of Saudi Arabia. In their study of the general Saudi adult population, they conducted a cross-sectional study and extended UTAUT with the construct of eHealth literacy. Similar to the findings of Alsahafi et al [34], this study found that performance expectancy and effort expectancy were positive predictors of behavioral intention. Contrary to our findings, social influence was found to be a positive predictor for behavioral intention to use a PHR in women. While gender, age, and internet experience were used as moderators, gender was the only variable with a significant moderating role in the aforementioned study. In contrast, our study found the experience with health apps to be the only significant moderator even though the moderating effect was small and accounted for 4.8% of the explained variance.

In the health care context, the integration of constructs from health behavior theories, such as perceived health threat and self-perception, may be useful [18,36]. Though UTAUT was developed to be a comprehensive framework to study technology acceptance, contextual considerations are required to explain PHR adoption behavior best.

### Implications for Practice

The Kingdom of Saudi Arabia has prioritized the use of eHealth technologies such as PHRs in health care delivery [34,35,37-39]. To meet the goals of the National Transformation Program, health care organizations around the country will increasingly be called upon to leverage PHRs to efficiently deliver person- and patient-centered care. This study may help organizations better understand patient perceptions of the PHR and lead them to identify strategies to engage patients with the PHR to better manage their health and well-being.

This study found that performance expectancy, effort expectancy, and attitude significantly impact the adoption of PHRs. Tailored marketing strategies have been used to promote the advantages of PHRs and are a way for patients to see the benefits of using a PHR to manage their health [7]. The design and functionalities of the PHR can play an important role in patients’ intention to use [7]. Designing a PHR with an easy-to-use, attractive interface with simple language will improve patients’ perceptions of the ease of use and help prevent health disparities [40]. Attitude have been identified as a barrier to the use of PHRs in a number of studies [7]. Patients may have negative attitudes toward a PHR for a number of reasons, and this can contribute to their refusal to use PHRs. When health care providers educate and train patients on the features, functionalities, and benefits of the PHR, a positive attitude will develop and facilitate acceptance. However, for health care providers to play this role, they must be knowledgeable about the benefits and purpose of a PHR.

### Limitations

There are several limitations to this study. First, this was secondary data analysis, and all constructs for the independent variables were single-item measures. This could have affected the reliability and validity of our findings. Most conceptual constructs are complex and multifaceted and, therefore, a single item may not be an “accurate, comprehensive, and reliable measurement” [41]. However, this was necessary to avoid the respondent burden. Second, a common method bias may be present since the independent variable and dependent variable were measured at a single point in time with only 1 data collection instrument. Finally, the generalizability may have been affected because the study was limited to 1 organization in the country.

### Recommendations for Future Research

Because this study was subject to common method bias, future researchers should examine the independent and dependent variables at different time points and with at least 2 different instruments. We were unable to secure access to either the system logs or patient records, but a future study may incorporate these types of data to minimize this bias.

Examining theories in new contexts advances theories and increases external validity [13,42]. Selecting constructs that explain the behavioral intention relationship should be context-based. In this study, the model tested explained 48.9% of the variance in behavioral intention, suggesting the inclusion of attitude was relevant and reasonable. However, other predictors may have improved the model. Future studies may consider adding other constructs shown to be influential in PHR adoption or, more broadly, eHealth adoption. Alaiad et al [36] recommend including constructs recognized as inhibitors of technology adoption as well as adding constructs related to health-related behavior.

The construct of privacy and security should be investigated. Studies showed that privacy and security concerns have a significantly negative effect on behavioral intention to use a PHR [7,8,43-45]. As opposed to technology such as e-banking, PHRs may be accessible to a wide range of health care personnel [46] as well as family members. Patients have raised concerns about identity theft and the possibility of their leaked health information limiting employment opportunities [46]. This study is one of the few to evaluate the moderating effect of variables on the relationship between the independent variables and behavioral intention to use a PHR. Most PHR studies have not assessed moderating or mediating effects [8]. The only significant moderating effect observed was experience with health apps on the relationship between social influence and behavioral intention. Other variables acting as either mediators or moderators may help enrich our understanding of PHR adoption within this context. Abd-Alrazaq et al [47] developed the Abd-Alrazaq Model to examine mediating, moderating, and moderated mediating effects on patients’ behavioral intention to use a PHR in England.

For the moderator of health status, a single self-reported health status item was used owing to its simplicity and to reduce the respondent burden; it has been found to be a valid and reliable measure of health status in high-income countries [48]. However, operationalizing health status in another way may have provided alternative findings. Future studies should measure health status through another method.

Further, future studies should consider using more mixed-methods approaches. In the systematic review of PHR use by Abd-Alrazaq [8], 88% of the studies were quantitative. Mixed-methods studies are suitable to develop multiple perspectives and a comprehensive understanding of PHR adoption. A qualitative approach alongside quantitative methods will provide deeper insight into the patient’s perspective.

Finally, more studies should evaluate the health care provider’s perspective of PHR adoption. The focus on a more engaged patient has been a paradigm shift in medicine [49]. Therefore, understanding health care provider perspectives is fundamental to the successful implementation, adoption, and continued use of a PHR [49,50]. Negative or indifferent attitudes among health care providers have been identified as a barrier to patient adoption [7]. Fears of increased workload, threats to autonomy, or upsetting patients are some concerns [50]. Addressing these concerns can lead to health care provider endorsement and subsequent patient adoption.

### Conclusions

The use of PHRs in the Kingdom of Saudi Arabia is relatively new and will continue to grow in line with Vision 2030 and the MNG-HA’s aim to be a center of excellence through the effective use of technology in health care delivery. This study extended the UTAUT model by adding the construct of attitude along with age, gender, experience, and health status as moderators. Our findings show that performance expectancy and effort expectancy had a significant positive effect on behavioral intention. This study provides evidence that attitude had a significant positive effect on behavioral intention to use a PHR. Additionally, the impact of experience with health apps as a moderator of social influence was supported in our study. These results can help the organization further understand ways to encourage and support patients in adopting PHRs.

## Abbreviations

MNG-HA: Ministry of National Guard Health Affairs
PHR: personal health record
UTAUT: Unified Theory of Acceptance and Use of Technology
VIF: variance inflation factor
